# Growth hormone secretagogue receptor and cannabinoid receptor type 1 intersection in the mouse brain

**DOI:** 10.1007/s00429-024-02876-3

**Published:** 2024-12-19

**Authors:** Camila Saenz, Gimena Fernandez, Ramiro Llovera, María J. Tolosa, Sonia Cantel, Jean-Alain Fehrentz, Kenneth Mackie, Lorenzo Leggio, Jeffrey Zigman, Pablo N. De Francesco, Mario Perello

**Affiliations:** 1 Laboratory of Neurophysiology of the Multidisciplinary Institute of Cell Biology [IMBICE, Argentine Research Council (CONICET) and Scientific Research Commission, Province of Buenos Aires (CIC-PBA), National University of La Plata], La Plata, Buenos Aires Argentina; 2 Institut des Biomolécules Max Mousseron, Univ Montpellier, CNRS, ENSCM, Montpellier, France; 3 Department of Psychological & Brain Sciences, Indiana University, Bloomington Indiana, USA; 4 Clinical Psychoneuroendocrinology and Neuropsychopharmacology Section, Translational Addiction Medicine Branch, Intramural Research Program and National Institute on Alcohol Abuse and Alcoholism Division of Intramural Clinical and Biological Research, National Institute on Drug Abuse, National Institutes of Health, Baltimore, MD USA; 5 Center for Hypothalamic Research and Division of Endocrinology & Metabolism, Department of Internal Medicine, University of Texas Southwestern Medical Center, Dallas, TX USA; 6 Department of Surgical Sciences, Functional Pharmacology and Neuroscience, University of Uppsala, Uppsala, Sweden

**Keywords:** GHSR, CB1R, Ghrelin, Co-distribution

## Abstract

The growth hormone secretagogue receptor (GHSR) and the cannabinoid receptor type 1 (CB1R) are G-protein coupled receptors highly expressed in the brain and involved in critical regulatory processes, such as energy homeostasis, appetite control, reward, and stress responses. GHSR mediates the effects of both ghrelin and liver-expressed antimicrobial peptide 2, while CB1R is targeted by cannabinoids. Strikingly, both receptors mediate their effects by acting on common brain areas and their individual roles have been well characterized. However, the potential for their co-expression in the same neuronal subsets remains largely unexplored. Here, we aim to map the cell populations where GHSR and CB1R might converge, hypothesizing that their co-expression in specific brain circuits could mediate integrated physiological responses. By utilizing two complementary labeling techniques—GHSR-eGFP mice and Fr-ghrelin labeling of GHSR+ cells—along with specific CB1R immunostaining, we sought to visualize and quantify potential areas of overlap. Also, we analyzed several cell RNA sequencing datasets to estimate the fraction of brain cells expressing both GPCRs and their phenotype. Our neuroanatomical studies revealed evident overlap of GHSR+ and CB1R+ signals in specific neuronal subsets mainly located in the cerebral cortex, hippocampus and the amygdala. Transcriptomic analysis revealed specific subsets of Ghsr+/Cnr1+ glutamatergic neurons in the hippocampus and amygdala, as well as different subtypes of Ghsr+/Cnr1+ neurons in the midbrain, hypothalamus, pons, and medulla. Thus, we revealed that GHSR and CB1R interact differentially across specific regions of the mouse brain, providing new insights into how these receptors' actions are integrated. Current findings may open new avenues for dual therapeutic interventions in metabolic disorders, obesity, and psychiatric conditions.

## Introduction

G-protein coupled receptors (GPCRs) constitute the largest family of cell surface receptors and play key roles in controlling most physiological functions (Venkatakrishnan et al. 2013). GPCRs modulate cellular activity through several mechanisms, encompassing not only canonical signaling pathways but also interactions with other proteins, including other GPCRs (Dale et al. 2022). In this context, the growth hormone secretagogue receptor (GHSR) has emerged as a paradigmatic GPCR (Cornejo et al. 2021a). GHSR is highly expressed in the brain, and is known to modulate GH secretion, appetite, glucose homeostasis, mood, among other functions (Müller et al. 2015). GHSR acts as the receptor for two gastrointestinal hormones: ghrelin and liver-expressed antimicrobial peptide 2 (LEAP2), and it also exhibits several ligand-independent actions, including constitutive activity and allosteric modulation of other GPCRs (Cornejo et al. 2021a). GHSR interacts with several other GPCRs, such as dopamine and serotonin receptors, among others (Schellekens et al. 2015; Hedegaard and Holst 2020). The crosstalk of GHSR with other GPCRs displays, sometimes, dramatic effects. For instance, GHSR forms heteromers with dopamine receptor type 2, and targeting the heteromers in mice abrogates the anorexigenic effects of the dopamine receptor type 2 agonist cabergoline (Kern et al. 2012). The cannabinoid receptor type 1 (CB1R) is the most abundant GPCR expressed in the brain, where it modulates neurotransmitter release, appetite, pain, mood, memory, among other physiological processes (Zou and Kumar 2018). It is activated by endocannabinoids, such as anandamide, and by phytocannabinoids, such as delta-9-tetrahydrocannabinol, the principal psychoactive compound of cannabis (Howlett and Abood 2017). CB1R can also interact with a variety of other GPCRs to modulate key physiological functions (Hilairet et al. 2003; Hojo et al. 2008; Oyagawa and Grimsey 2021). Indeed, heteromers of CB1R with other GPCR, such as dopamine receptor type 2, have been proposed as a putative target to treat some neurological human conditions (Albizu et al. 2010). However, the use of novel GPCR ligands for clinical applications has remained limited, primarily due to emerging concerns stemming from adverse effects observed in some cases, such as the CB1R inverse agonist rimonabant (Sam et al. 2011). Thus, studying the crosstalk between GPCRs may have important implications not only for understanding the molecular basis mediating the actions of these receptors but also for the development of novel pharmacotherapies.

Some evidence suggests a crosstalk between GHSR and CB1R. Both receptors use common signal transduction pathways and play similar functions in regulating appetite, body weight, stress-related behaviors, antidepressant-like and rewarding processes (Edwards and Abizaid 2016). Studies in rodents revealed that the action of GHSR involves CB1R activity. For instance, ghrelin fails to induce food intake in CB1R-knock-out mice and in rodents treated with either rimonabant or other CB1R antagonists (Tucci et al. 2004; Kola et al. 2008; Alen et al. 2013; Senin et al. 2013; Ting et al. 2015). Also, intra-ventral tegmental area (VTA)-injected ghrelin does not stimulate locomotor activity in mice pretreated with rimonabant (Kalafateli et al. 2018), and ghrelin-induced GH secretion is abrogated by systemic treatment with rimonabant in rats (Al-Massadi et al. 2010; Kola et al. 2013). Conversely, CB1R activation does not recruit its canonical intracellular signaling pathways in the hypothalamus, visceral fat or liver of GHSR-knock out mice (Lim et al. 2013). Furthermore, CB1R blockage was shown to reduce alcohol intake, in part, by reducing ghrelin synthesis in the stomach (Godlewski et al. 2019).

Notably, previous neuroanatomical studies investigating the independent expression patterns of GHSR and CB1R have shown that they are often expressed in similar areas of the mouse brain (Zigman et al. 2006; Mani et al. 2014). In cultures of striatal neurons, they are even expressed in the same cells and the existence of GHSR and CB1R complexes was suggested by *in situ* proximity ligation imaging assays (Lillo et al. 2021). Strikingly, however, we were unable to find any neuroanatomical analysis of their simultaneous presence in the mouse brain that could clarify the putative crosstalk between these GPCRs. To determine the potential neuronal subsets where the actions of both receptors could converge, we simultaneously examined the distribution of GHSR+ cells and CB1R immunoreactivity throughout the adult mouse brain. Specifically, we visualized the presence of CB1R using a validated anti-CB1R antibody (Hájos et al. 2000), whereas cells expressing GHSR were studied using two complementary approaches: (1) a mouse line that expresses enhanced green fluorescent protein (eGFP) under the GHSR promoter (GHSR-eGFP mice) (Mani et al. 2014), and (2) a labeling strategy using a fluorescent variant of ghrelin (Fr-ghrelin) (Cabral et al. 2013; Uriarte et al. 2021). Furthermore, we estimated the fraction and the phenotype of cells expressing both GPCRs in different regions of the mouse brain using different single-cell RNA sequencing datasets.

## Materials and methods

### Mice

This study was conducted in adult (9–12 weeks old) wild-type (WT) and GHSR-eGFP mice that were in a pure C57BL/6 background (Mani et al. 2014). All experimental mice were maintained under controlled conditions (22±1°C, 12-h light cycle from 7:00 am to 7:00 pm) with regular chow diet and water available *ad libitum*. We used: 5 WT mice (3 female and 2 male) and 6 GHSR-eGFP mice (4 female and 2 male). The protocols were approved by the Institutional Animal Care and Use Committee of the IMBICE.

### Labeling with Fr-ghrelin

Fr-ghrelin, a fluorescent variant of ghrelin conjugated to DY-647P1 through a C-terminal Cys (Uriarte et al. 2021), was intracerebroventricularly (ICV) injected into WT mice during stereotaxic surgeries. Briefly, anaesthetized mice were stereotaxically implanted with a single indwelling guide ICV cannula (4-mm long, 22-gauge, PlasticsOne) in the lateral ventricle using the following coordinates: -0.34 mm antero-posterior; ±1 mm medio-lateral and -2.3 mm dorso-ventral. Then, mice were ICV-injected with 2 uL containing 60 pmol/mouse of Fr-ghrelin, which is the minimum amount of fluorescent analog that can be directly visualized in fixed coronal brain slices. ICV injections were made over 2 min through a 30-gauge needle that extended 0.5 mm below the guide cannula. The injector cannula was left in place for 2 min following the injection to prevent backflow of the injected solution. Fr-ghrelin was allowed to diffuse for 30 min. The correct location of the ICV cannulas was confirmed by histological observation at the end of the experiment.

### Brain tissue processing

Anaesthetized WT mice ICV-injected with Fr-ghrelin and GHSR-eGFP mice were transcardiacally perfused with heparinized phosphate-buffered saline (PBS, pH = 7.4) and formaldehyde (4% in PBS). Next, brains were removed, post-fixed in the same fixative solution for 2 h and incubated with sucrose (20% in PBS) overnight. Next, brains were frozen and coronally sectioned into four equal series of 40-μm thick sections on a cryostat. Sections were stored at −20 °C in a cryoprotective solution containing 30% ethylene glycol in PBS until immunolabeling.

### Double immunolabeling

Coronal brain sections of WT mice ICV-injected with Fr-ghrelin and GHSR-eGFP mice were immunolabeled using an affinity-purified rabbit serum against CB1R (Mackie laboratory, Indiana University, Cat# rCB1-CT, 1:750, RRID: AB_2315249). The antiserum was generated using the last 73 amino acid residues of the cloned rat CB1R fused to glutathione S-transferase, as the antigen (Wager-Miller et al. 2002). Briefly, one series of coronal sections was blocked with adult horse serum (2% in Triton X-100 0.25% in PBS) for 1 h, and then incubated with either a rabbit anti-CB1R serum alone in samples of WT mice ICV-injected with Fr-ghrelin or a rabbit anti-CB1R serum with a goat anti-eGFP antibody (Vector Lab., Cat# BA-0702, 1:1000, RRID: AB_2336121) in samples of GHSR-eGFP mice for 48 h at 4°C. Then, sections were washed and incubated for 2 h at room temperature with either a donkey anti-rabbit Alexa Fluor 594 antibody (Thermo Fisher, Cat# A-21207, 1:1000, RRID: AB_141637) alone or in a combination with a donkey anti-goat Alexa Fluor 488 (Thermo Fisher, Cat# A-11055, 1:1000, RRID: AB_2534102) antibody for brain sections of WT mice ICV-injected with Fr-ghrelin or GHSR-eGFP mice, respectively. Finally, brain sections were washed and mounted on glass slides and coverslipped with mounting media containing Hoechst to visualize cell nuclei.

### Quantitative neuroanatomical analysis

**Fig. 1:**
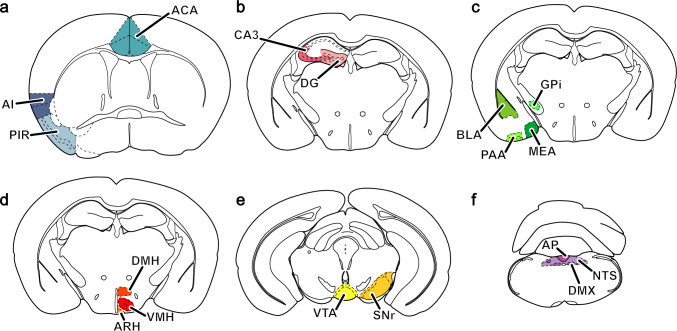
Schematic representation of the brain areas analyzed in the current study. **a** Cerebral cortex: anterior cingulate area (ACA); agranular insular area, posterior part (AIp); piriform area (PIR). **b** Hippocampal formation, HPF: dentate gyrus (DG); field CA3 (CA3). **c** Amygdala: basolateral amigdalar nucleus (BLA), piriform amygdalar area (PAA), medial amygdalar nucleus (MEA) and globus pallidus, internal segment (GPi). **d** Hypothalamus, HY: arcuate hypothalamic nucleus (ARH); ventromedial hypothalamic nucleus (VMH); dorsomedial hypothalamic nucleus (DMH). **e** Midbrain, MB: Substantia nigra, reticular part (SNr); ventral tegmental area (VTA). **f** Medulla, MY: area postrema (AP); dorsal motor nucleus of the vagus nerve (DMX), nucleus of the solitary tract (NTS)

**Fig. 2:**
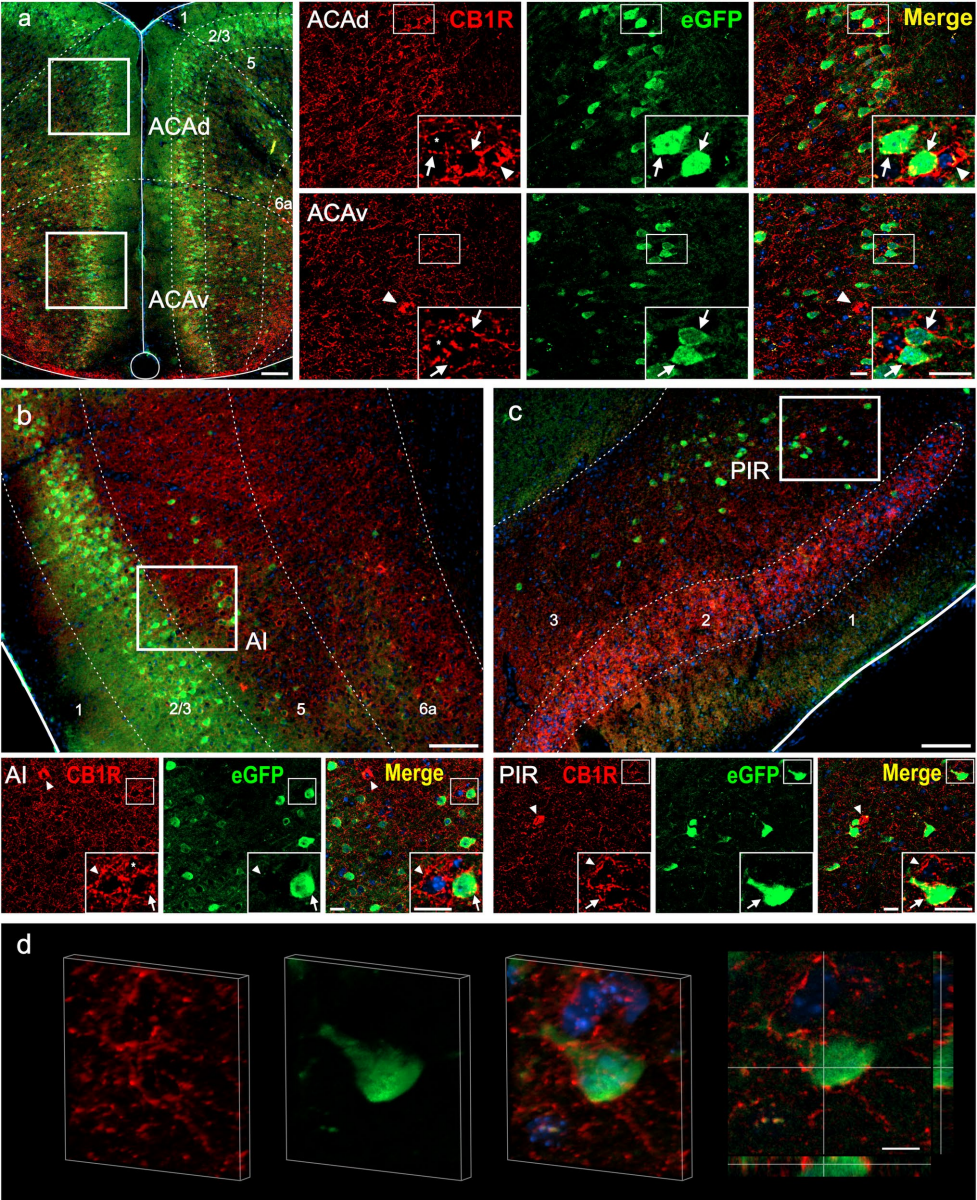
CB1R+ and eGFP+ signals in the cerebral cortex of GHSR-eGFP mice. Representative microphotographs of brain coronal sections of GHSR-eGFP mice subjected to double fluorescent immunostaining against CB1R (red) and eGFP (green) showing: **a** anterior cingulate area, dorsal and ventral part (ACAd and ACAv), **b** agranular insular area (AI) and **c** piriform area (PIR). Cortical cells are arranged in layers denoted as 1, 2, 3, 5, 6a. Cell nuclei were labeled with Hoechst (blue). **d** and **e** 3D-rendering and orthogonal views of image stacks corresponding to two of the cells from panel c (cell shown in the inset and cell marked with a dotted square, respectively). Both display a typical perisomatic CB1R+ signal. Renderings are shown for the merge of CB1R+ (red), eGFP (green) and Hoechst stain (blue). Sectioning planes are indicated with gray lines in the orthogonal views. The volumetric renderings were generated using the Fiji plugin 3Dscript (Schmid et al. 2019). Insets show high magnification images of the area marked in the low magnification image. Arrows mark cells showing double eGFP+ and CB1R+ signals. Asterisks indicate CB1R+ signal with a dense meshwork-like pattern. Arrowheads point at intensely stained CB1R+ cell bodies that lack eGFP+ signal. Scale bars: 100 µm (low magnification image), 20 µm (high magnification insets) and 10 μm (orthogonal views)

Fluorescence images were acquired with 10x/0.45 (low magnification) and 40×/0.95 (high magnification) air objectives using a Zeiss AxioObserver D1 equipped with an Apotome.2 structured illumination module and an AxioCam 506 monochrome camera. All image processing and analysis were performed in the ImageJ-based open-source image-processing package Fiji (Schindelin et al. 2012). The neuroanatomical analysis was performed in specific areas of the cerebral cortex, hippocampus, amygdala, hypothalamus, midbrain, and medulla oblongata, as shown in Fig. 1. The neuroanatomical limits of each brain region were identified using a mouse brain atlas (Paxinos and Franklin 2001). Brain slices of Fr-ghrelin-injected WT mice were used to analyze Fr-ghrelin positive (Fr-ghrelin+) and CB1R positive (CB1R+) signals, whereas brain slices of GHSR-eGFP mice were used to analyze eGFP positive (eGFP+) and CB1R+ signals. Overlapping signals were quantified in 40X images of representative anterior, medial and posterior coronal sections for each brain area. A unique average of the fraction of double-labeled CB1R+/eGFP+ or CB1R+/Fr-ghrelin+ cells in each brain region of each mouse was calculated using, at least, three coronal sections and expressed as a percentage of the total number of eGFP+ or Fr-ghrelin+ cell bodies, respectively. Finally, the mean ± standard error of the mean (SEM) per brain area was estimated for each experimental strategy using pooled male and female datasets (sample size for pooled data: n=5 for WT and n=6 for GHSR-eGFP mice, for all brain regions). The volumetric renderings shown in Fig. 2 were generated using the Fiji plugin 3Dscript (Schmid et al. 2019).

### Transcriptomics

To analyze the abundance, co-distribution and phenotype of *Ghsr*- and *Cnr1*-expressing cells, we used a high-resolution transcriptomic and spatial cell-type atlas from the mouse brain generated at the Allen Institute for Brain Science (Yao et al. 2023). This atlas is comprised of a 10x Chromium scRNAseq dataset from microdissections covering the whole brain (~4.0 million cells) and a spatial transcriptomic MERFISH dataset generated from 10 μm coronal sections sampled at 200-μm intervals from a single male mouse (~4.3 million cells), with panel of 500 genes and registered to the Allen Mouse Brain Common Coordinate Framework (CCFv3). The metadata corresponding to both datasets was used as reported by the authors, including UMAP coordinates, cell-type and anatomical annotations (release 20240831). The latest release of these processed and annotated datasets can be accessed at: https://alleninstitute.github.io/abc_atlas_access/descriptions/WMB_dataset.html. The 10x dataset was used as the primary reference, as it contained both genes of interest. *Cnr1*- and *Ghsr*-expressing cells (Cnr1+ and Ghsr+ cells, respectively) were defined as those having a log2(CPM) expression higher than 3 for both genes. The distribution of single and double-positive Ghsr+/Cnr1+ cells in the different structures mouse brain was estimated by leveraging the shared hierarchical clustering between datasets. First, the proportion of positive cells was computed for all clusters in the 10x set. Then, the proportion of clusters present in each structure was determined in the MERFISH set. Finally, the number of positive cells was apportioned per cluster for each structure, rounded to the integer part, and summarized, resulting in a list of estimated positive counts by structure for the spatial dataset. These estimates were refined by correcting for the partial volume of some structures due to missing sections, and scaled-up for a whole brain estimate by multiplying with a proportionality factor obtained by regression between estimated cell counts and literature reports for different hypothalamic cell populations: *Agrp*+ in the ARH (Betley et al. 2013); *Pomc*+, *Npy*+ and *Gfap*+ in the ARH (Lemus et al. 2015); and *Oxt*+, *Avp*+ and *Crh*+ in the PVH (Biag et al. 2012). A list of the most prominent structures with double-positive cells was generated by selecting those having more than 350 double-positive cells per structure and a density of more than 50 cells per mm^3^.

In addition, single-cell (sc) RNA-seq datasets from 1) cortex and hippocampal regions (Yao et al. 2021), in which cortex included isocortex and entorhinal cortex and the hippocampal region includes the hippocampus as well as the prosubiculum proper (Sub), the prosubiculum (ProS), the parasubiculum/postsubiculum/presubiculum (PPP), the intratelencephalic region (IT), the retrohippocampal region (RHP) and layer 2/3 (L2/3); from 2) whole hypothalamus (Steuernagel et al. 2022), which comprises a set of 18 publicly available datasets; and from 3) the area postrema (Zhang et al. 2021) were analyzed. These original datasets can be accessed here: https://portal.brain-map.org/atlases-and-data/rnaseq/mouse-whole-cortex-and-hippocampus-10x, https://doi.org/10.17863/CAM.87955 and https://github.com/jakaye/AP_scRNA. Here, Cnr1+ and Ghsr+ cells were defined as the cells that have one or more counts in the transcript count matrix without any previous normalization. Metadata for cells including taxonomy (clusters) and UMAP coordinates was used as defined and reported by the authors.

Genes of interest from the whole mouse brain dataset were extracted from the expression matrices using the AnnData library v0.10.8 with Python 3.9.19. In all cases, integration of expression matrices with the corresponding metadata and further processing was performed in R 4.4.1/RStudio 2024.04.2+764.

## Results

### CB1R immunolabeling in GHSR*-eGFP* mice

**Fig. 3:**
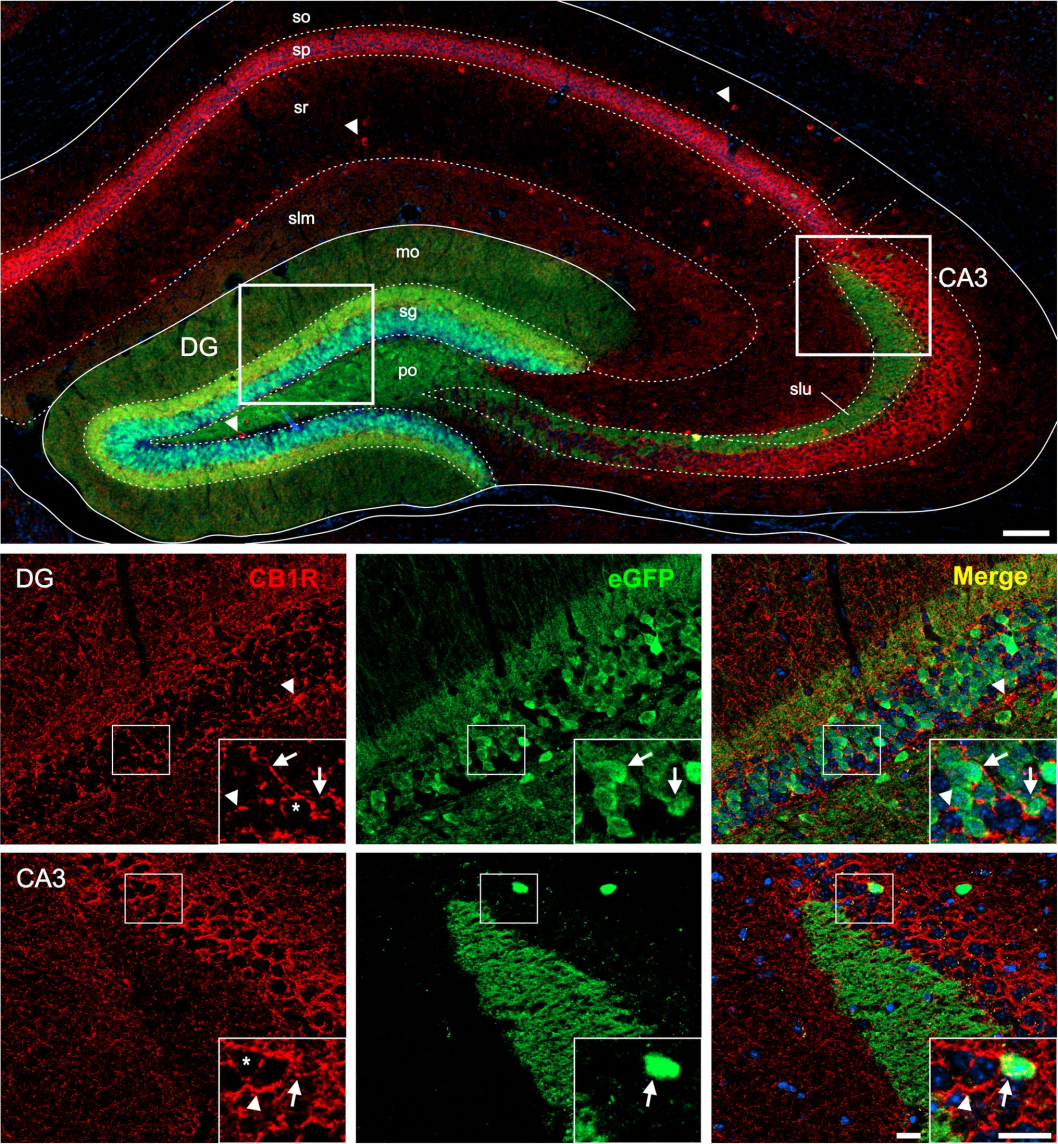
CB1R+ and eGFP+ signals in the hippocampus of GHSR-eGFP mice. Representative microphotographs of brain coronal sections of GHSR-eGFP mice subjected to double fluorescent immunostaining against CB1R (red) and eGFP (green) showing dentate gyrus (DG) and field CA3 (CA3). DG cells are arranged in layers denoted as molecular layer (mo), polymorphic layer (po), granular cell layer (sg). CA3 cells are arranged in layers denoted as stratum lacunosum moleculare (slm), stratum lucidum (slu), stratum oriens (so), pyramidal layer (sp), stratum radiatum (sr). Cell nuclei were labeled with Hoechst (blue). Insets show high magnification images of the area marked in the low magnification image. Arrows mark cells showing double eGFP+ and CB1R+ signals. Asterisks indicate CB1R+ signal with a dense meshwork-like pattern. Arrowheads point at intensely stained CB1R+ cell bodies that lack eGFP+ signal. Scale bars: 100 µm (low magnification image) and 20 µm (high magnification images)

**Fig. 4:**
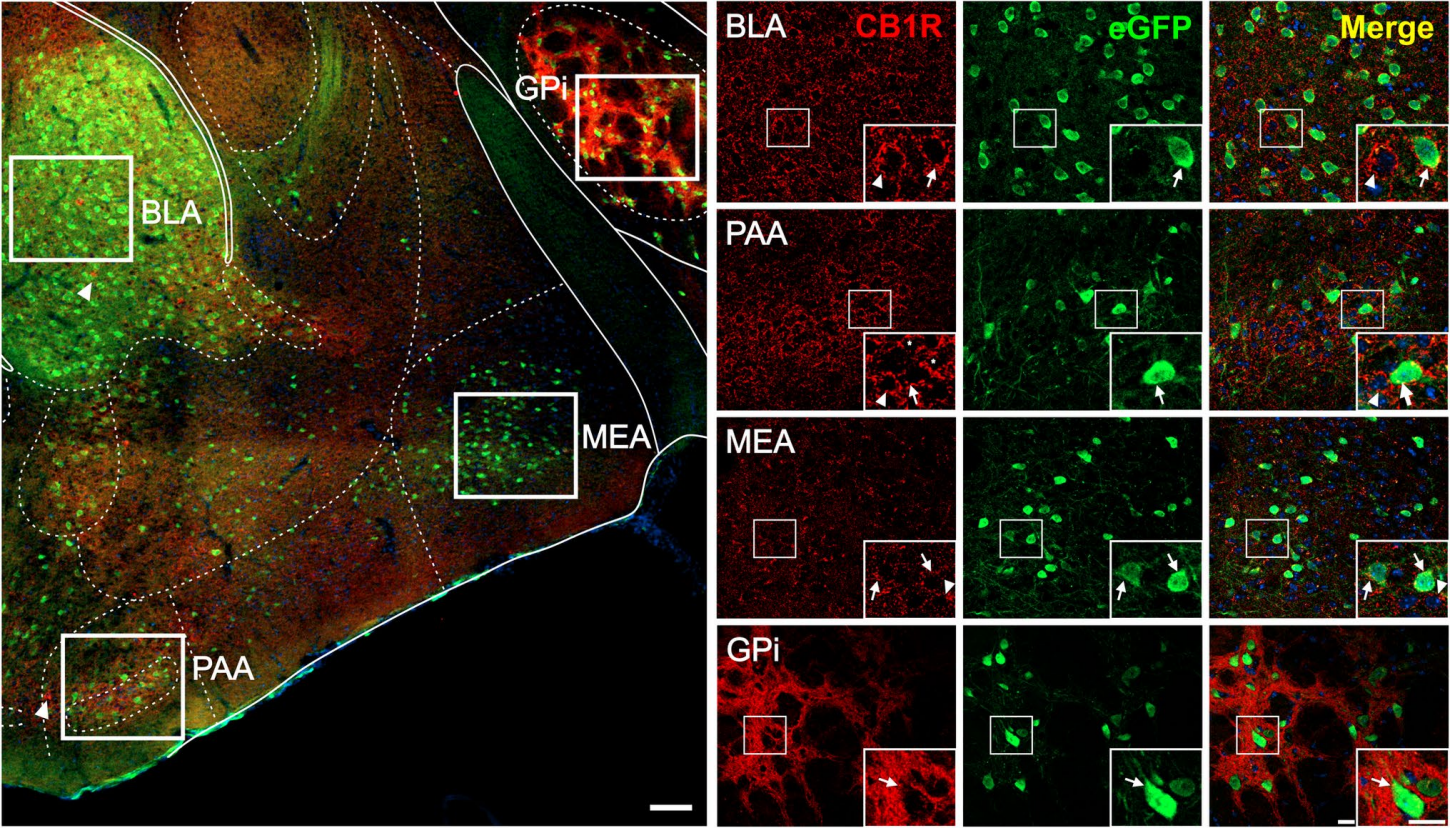
CB1R+ and eGFP+ signals in the amygdala of GHSR-eGFP mice. Representative microphotograph of brain coronal section of GHSR-eGFP mice subjected to double fluorescent immunostaining against CB1R (red) and eGFP (green) showing basolateral amigdalar nucleus (BLA), piriform amygdalar área (PAA), medial amygdalar nucleus (MEA) and globus pallidus, internal segment (GPi). Cell nuclei were labeled with Hoechst (blue). Insets show high magnification images of the area marked in the low magnification image. Arrows mark cells showing double eGFP+ and CB1R+ signals. Asterisks indicate CB1R+ signal with a dense meshwork-like pattern. Arrowheads point at intensely stained CB1R+ cell bodies that lack eGFP+ signal. Scale bars: 100 µm (low magnification image) and 20 µm (high magnification images)

**Fig. 5:**
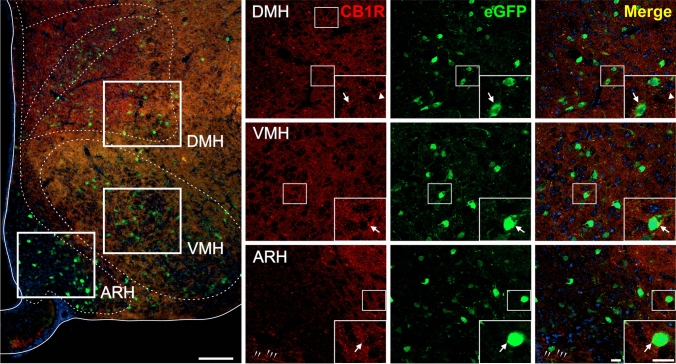
CB1R+ and eGFP+ signals in the hypothalamus of GHSR-eGFP mice. Representative microphotograph of brain coronal section of GHSR-eGFP mice subjected to double fluorescent immunostaining against CB1R (red) and eGFP (green) showing arcuate hypothalamic nucleus (ARH); ventromedial hypothalamic nucleus (VMH); dorsomedial hypothalamic nucleus (DMH). Cell nuclei were labeled with Hoechst (blue). Insets show high magnification images of the area marked in the low magnification image. Arrows mark cells showing double eGFP+ and CB1R+ signals. Arrowheads point at stained CB1R+ signal that lack eGFP+ signal. Scale bars: 100 µm (low magnification image) and 20 µm (high magnification images)

**Fig. 6:**
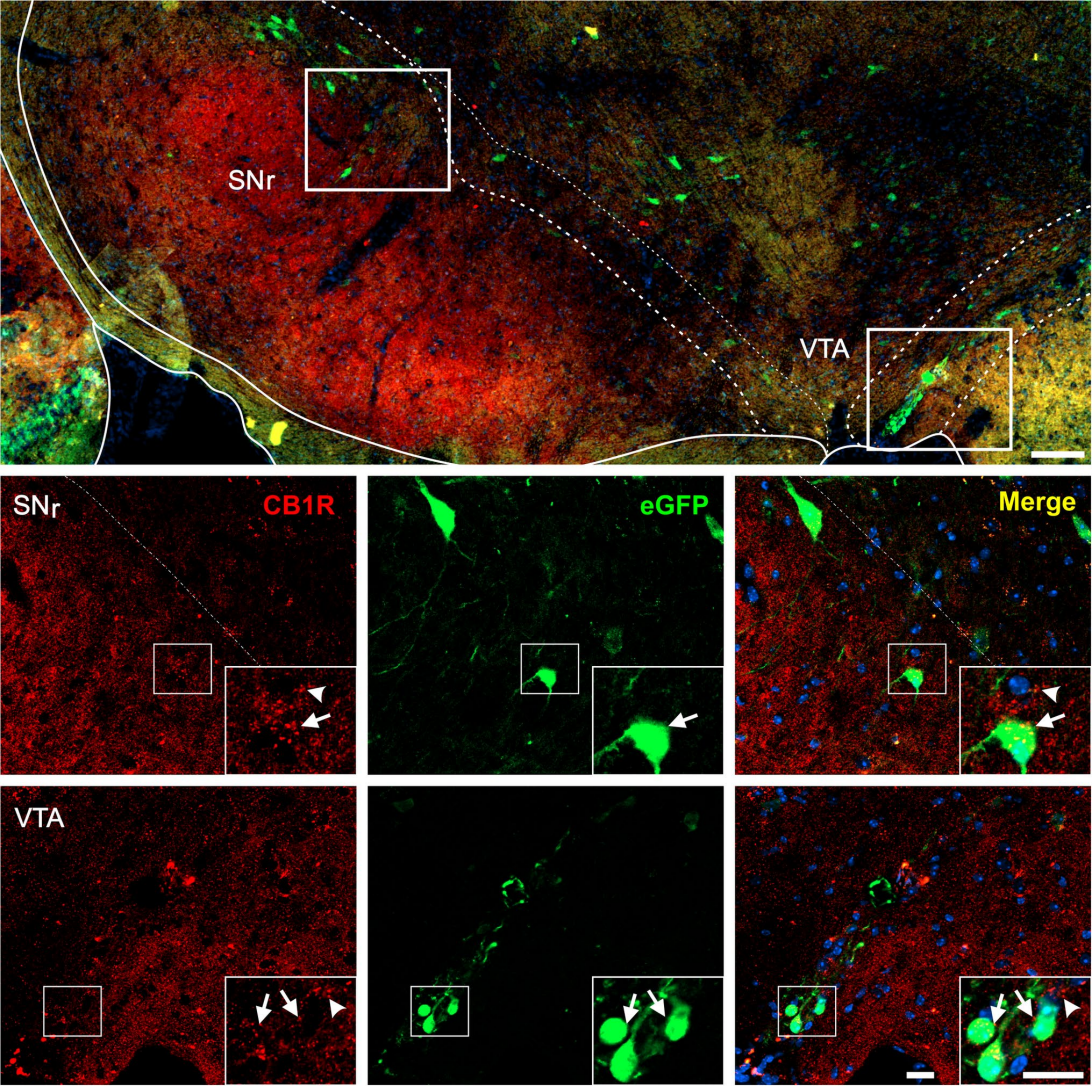
CB1R+ and eGFP+ signals in the midbrain of GHSR-eGFP mice. Representative microphotograph of brain coronal section of GHSR-eGFP mice subjected to double fluorescent immunostaining against CB1R (red) and eGFP (green) showing substantia nigra, reticular part (SNr) and ventral tegmental area (VTA). Cell nuclei were labeled with Hoechst (blue). Insets show high magnification images of the area marked in the low magnification image. Arrows mark cells showing double eGFP+ and CB1R+ signals. Arrowheads point at stained CB1R+ signal that lack eGFP+ signal. Scale bars: 100 µm (low magnification image) and 20 µm (high magnification images)

**Fig. 7:**
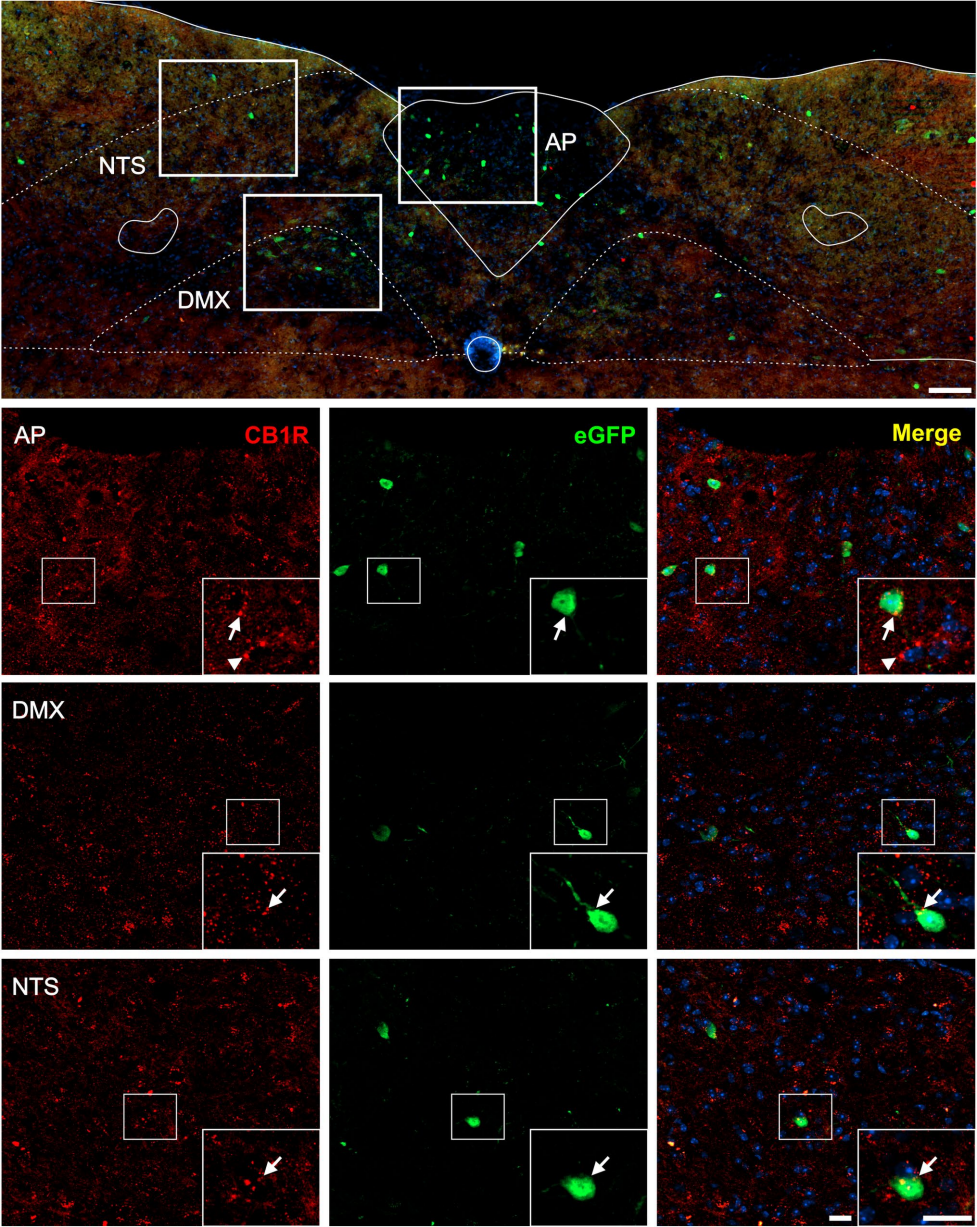
CB1R+ and eGFP+ signals in the medulla oblongata of GHSR-eGFP mice. Representative microphotograph of brain coronal section of GHSR-eGFP mice subjected to double fluorescent immunostaining against CB1R (red) and eGFP (green) showing area postrema (AP), dorsal motor nucleus of the vagus nerve (DMX) and nucleus of the solitary tract (NTS). Cell nuclei were labeled with Hoechst (blue). Insets show high magnification images of the area marked in the low magnification image. Arrows mark cells showing double eGFP+ and CB1R+ signals. Arrowheads point at stained CB1R+ signal that lack eGFP+ signal. Scale bars: 100 µm (low magnification image) and 20 µm (high magnification images).

First, we performed double immunolabeling for CB1R and eGFP on coronal brain sections of GHSR*-eGFP* mice and confirmed that CB1R+ and eGFP+ signals were found in the same brain regions as has been previously described for each signal separately (Tsou et al. 1998; Mani et al. 2014). The eGFP+ signal was mainly found in cell bodies but also in some fibers, whereas CB1R+ signal depicted a polymorphic pattern depending on the brain area. We performed systematic quantitative assessments of labeling patterns in both sexes and did not observe any evident differences between them (data not shown). Therefore, we pooled the male and female data to increase the sample sizes of our estimates. Figures 2, 3, 4, 5, 6, 7 show representative photomicrographs of eGFP+ and CB1R+ signals in different brain regions (see below).

In the cerebral cortex (Fig. 2), eGFP+ signal was found in cell bodies which formed a laminar distribution and were mainly packed in the granular layer (layer 2/3), but were also present in non-granular layers 5 and 6a. eGFP+ cell bodies were located in most of the cortical regions, including the anterior cingulate area, dorsal and ventral part (ACAd and ACAv, respectively, Fig. 2a), the agranular insular area (AI, Fig. 2b), the piriform area (PIR, Fig. 2c), the prelimbic area (PL, not shown) and the ectorhinal area (ECT, not shown). CB1R+ signal was ubiquitously found mainly as a meshwork-like pattern, but also labeling some cell bodies. Analysis of the double immunolabeling revealed that a fraction of the eGFP+ cell bodies had overlapping CB1R+ signal, which was perisomatically distributed around the eGFP+ cell bodies, as shown in the representative examples in Fig. 2d, e. Quantitative analysis indicated that 72.1±3.7, 59.6±2.2 and 68.0±8.6 % of eGFP+ cell bodies presented overlapping CB1R+ signal in the ACAd, AI and PIR, respectively. Conversely, fully stained CB1R+ cell bodies did not overlap with eGFP+ signal in these cortical regions.

In the hippocampus, eGFP+ signal was mainly found in the dentate gyrus (DG), where it labels cell bodies clustered in the base of the granular layer (sg) and fibers in the molecular (mo) and in the polymorphic (po) layers (Fig. 3). The eGFP+ signal was also found in all subfields of the Cornu Ammonis (CA), especially in the subfield 3 (CA3). In particular, eGFP+ signal was observed in fibers in the stratum lucidum (slu) and in cell bodies scattered across the pyramidal layer (sp). On the other hand, CB1R+ signal was enriched in the sp of the CA region, where it displayed a dense meshwork-like pattern in which eGFP+ cell bodies were intermingled. Also, evident CB1R+ cell bodies were observed scattered throughout the stratum oriens (so) and stratum radiatum (sr) of the CA as well as in the interface between the sg and po of the DG. In the DG, CB1R+ signal displayed a loose meshwork-like pattern clustered in the top of the DG-sg that contained interspersed eGFP+ cell bodies. The simultaneous analysis of both signals revealed that 89.9±8.1 % of the eGFP+ cell bodies of the CA3 have overlapping CB1R+ signal, which was also perisomatically distributed. Conversely, 53.8±2.7 % eGFP+ cell bodies of the DG had overlapping CB1R+ signal. CB1R+ cell bodies of the CA did not overlap with eGFP+ signal.

A high number of eGFP+ cell bodies was observed in the amygdala (Fig. 4), particularly in the basolateral amygdalar nucleus (BLA). Less intense eGFP+ cell bodies were also observed in other sub-nuclei of the amygdala, including the medial amygdalar nucleus (MEA) and the piriform amygdalar area (PAA). CB1R+ signal in the amygdala presented a polymorphic pattern which included evident CB1R+ cell bodies scattered thought the BLA and PAA. CB1R+ signal in the amygdala also included a loose meshwork-like pattern mainly found in the BLA and PAA, as well as a sparse punctate pattern in the MEA. The simultaneous analysis of both signals revealed that 40.8±9.1 and 69.9±7.6 % of eGFP+ cell bodies in the PAA and BLA, respectively, contain overlapping CB1R+ signal, in a similar pattern as described in the cortical areas. Conversely, CB1R+ cell bodies lacked eGFP+ signal. At this coronal level, eGFP+ cell bodies were also found in the globus pallidus, internal segment (GPi), where CB1R+ signal was found as a dense punctate signal that was traversed by unlabeled fascicles and surrounded eGFP+ cell bodies.

In the hypothalamus (Fig. 5), eGFP+ cell bodies were found in all the hypothalamic nuclei as previously described (Mani et al. 2014), including the arcuate (ARH), the dorsomedial (DMH) and the ventromedial (VMH). Here, the CB1R+ signal was markedly weaker than in other brain areas, such as the hippocampus, and it was exclusively found in a sparse punctate pattern. The CB1R+ signal was uniformly distributed in DMH, VMH and the lateral ARH, but not in the medial ARH near the median eminence, where it was more sporadic and displayed a beads-on-a-string pattern. The analysis of double immunolabeling in the ARH and other hypothalamic nuclei indicated that CB1R+ signal was occasionally found overlapping on the peri-somatic region of the eGFP+ cell bodies.

In the brainstem, eGFP+ and CB1R+ signals were also found as previously reported (Tsou et al. 1998; Mani et al. 2014). In the midbrain, eGFP+ cell bodies were sporadically detected in several areas including the periaqueductal gray (PAG), the compact and lateral regions of the substantia nigra, reticular part, (SNr) as well as in the parabrachial pigmented (PBP) and paranigral (PN) sub-nuclei of the VTA. In contrast, CB1R+ signal in the midbrain displayed a punctate pattern that was more densely enriched in the SNr compared to other midbrain areas (e.g., VTA, PAG). Analysis of double labeling indicated that the vast majority of eGFP+ cell bodies depicted overlapping CB1R+ signal, especially in the SNr (Fig. 6). In the medulla oblongata, eGFP+ cell bodies were found in the area postrema (AP), the dorsal motor nucleus of the vagus (DMX) and the nucleus of the solitary tract (NTS). Here, CB1R+ signal was also rather weak and displayed a sparse punctate pattern (Fig. 7). In all three nuclei of the dorsal vagal complex, some eGFP+ cell bodies showed small amounts of overlapping punctate CB1R+ signal.

### CB1R immunolabeling in WT mice centrally-injected with Fr-ghrelin

**Fig. 8:**
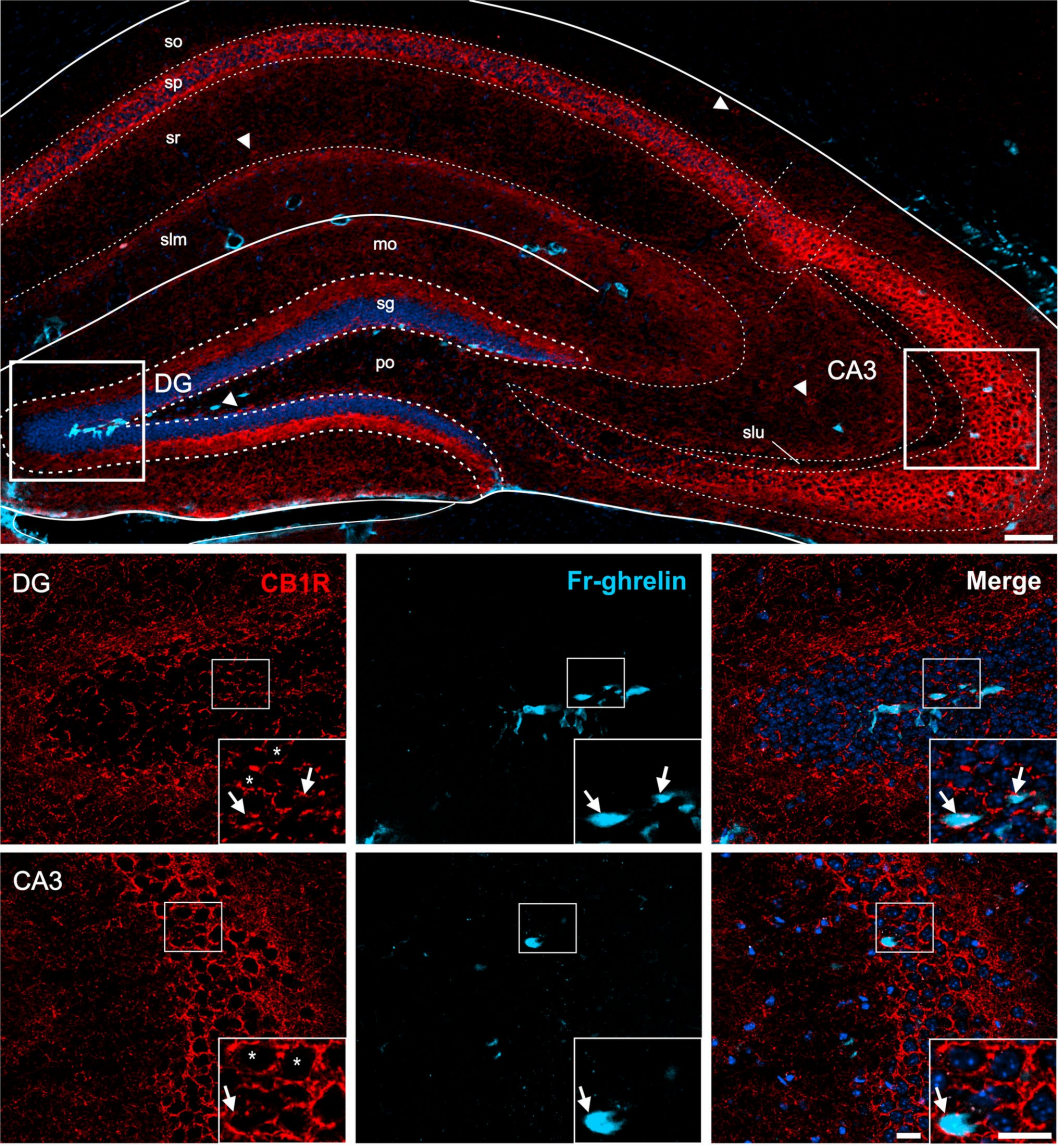
CB1R+ and Fr-ghrelin signals in the hippocampus of WT mice centrally-injected with the fluorescent ghrelin analog. Representative microphotograph of brain coronal section of WT mice ICV-injected with Fr-ghrelin (cyan) subjected to fluorescent immunostaining against CB1R (red) showing dentate gyrus (DG) and field CA3 (CA3). DG cells are arranged in layers denoted as molecular layer (mo), polymorphic layer (po), granular cell layer (sg). CA3 cells are arranged in layers denoted as stratum lacunosum moleculare (slm), stratum lucidum (slu), stratum oriens (so), pyramidal layer (sp), stratum radiatum (sr). Cell nuclei were labeled with Hoechst (blue). Insets show high magnification images of the area marked in the low magnification image. Arrows mark cells showing double Fr-ghrelin+ and CB1R+ signals. Asterisks indicate CB1R+ signal with a dense meshwork-like pattern. Arrowheads point at intensely stained CB1R+ cell bodies that lack Fr-ghrelin+ signal. Scale bars: 100 µm (low magnification image) and 20 µm (high magnification images)

**Fig. 9:**
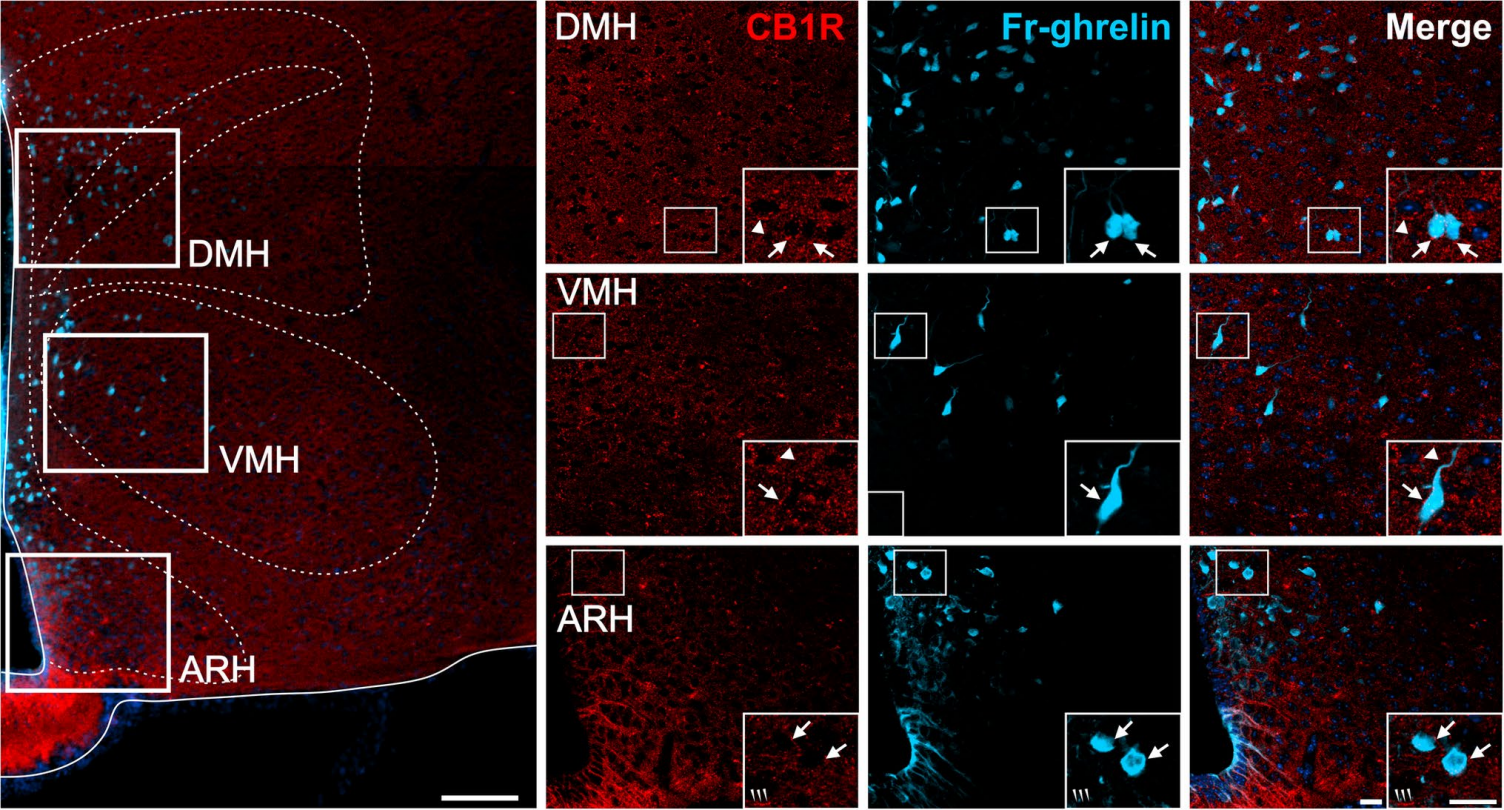
CB1R+ and Fr-ghrelin signals in the hypothalamus of WT mice centrally-injected with the fluorescent ghrelin analog. Representative microphotograph of brain coronal section of of WT mice ICV-injected with Fr-ghrelin (cyan) subjected to fluorescent immunostaining against CB1R (red) showing arcuate hypothalamic nucleus (ARH); ventromedial hypothalamic nucleus (VMH); dorsomedial hypothalamic nucleus (DMH). Cell nuclei were labeled with Hoechst (blue). Insets show high magnification images of the area marked in the low magnification image. Arrows mark cells showing double Fr-ghrelin+ and CB1R+ signals. Arrowheads point at stained CB1R+ signal that lack Fr-ghrelin+ signal. Scale bars: 100 µm (low magnification image) and 20 µm (high magnification images)

**Fig. 10:**
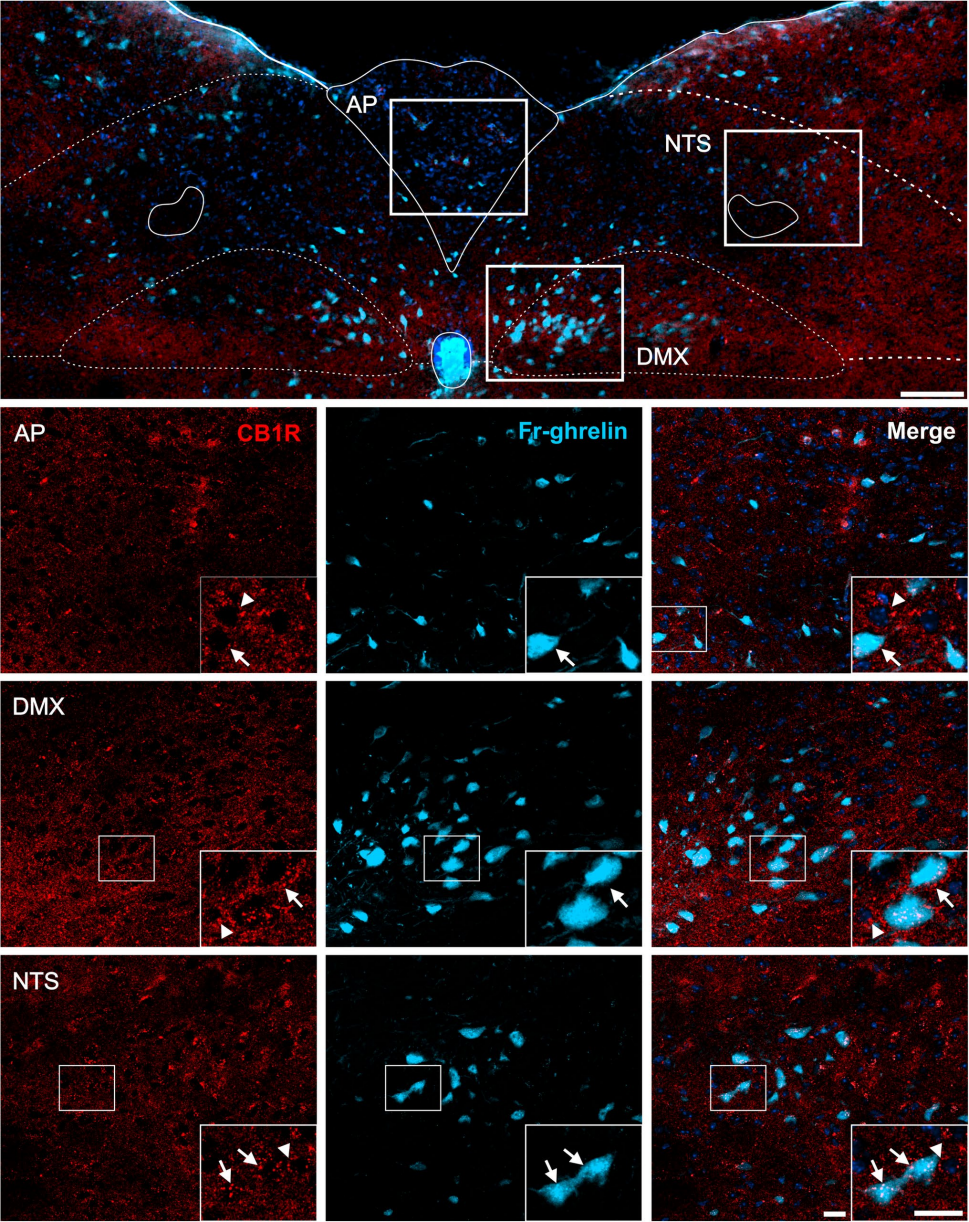
CB1R+ and Fr-ghrelin signals in the medulla oblongata of WT mice centrally-injected with the fluorescent ghrelin analog. Representative microphotograph of brain coronal section of WT mice ICV-injected with Fr-ghrelin (cyan) subjected to fluorescent immunostaining against CB1R (red) showing area postrema (AP), dorsal motor nucleus of the vagus nerve (DMX) and nucleus of the solitary tract (NTS). Cell nuclei were labeled with Hoechst (blue). Insets show high magnification images of the area marked in the low magnification image. Arrows mark cells showing double Fr-ghrelin+ and CB1R+ signals. Arrowheads point at stained CB1R+ signal that lack Fr-ghrelin+ signal. Scale bars: 100 µm (low magnification image) and 20 µm (high magnification images)

As a complementary strategy to assess the brain areas where GHSR and CB1R could simultaneously act, we performed immunolabeling against CB1R in brain sections of WT mice ICV-injected with Fr-ghrelin. In this case, the Fr-ghrelin+ signal was exclusively found in cell bodies localized in regions adjacent to brain ventricles, as previously shown (Cabral et al. 2013), while the CB1R+ signal displayed a similarly polymorphic pattern, depending on the brain area, as described above. In the hippocampus, Fr-ghrelin+ cell bodies were mainly observed in the base of the sg of the DG, and sporadically found in the sp of the CA. Quantitative analysis revealed that 38.4±3.0 % of Fr-ghrelin+ cell bodies in the DG had overlapping CB1R+ signal, whereas 80.0±6.1 % of the Fr-ghrelin+ cell bodies in the CA3 had overlapping CB1R+ signal, which was perisomatically distributed (Fig. 8). In the hypothalamus, Fr-ghrelin+ cell bodies were found in the periventricular regions of the ARH, the VMH and the DMH, and they showed occasional and sparse CB1R+ signal overlapping in the peri-somatic area (Fig. 9). In the AP, the DMX and the NTS of the medulla oblongata, some Fr-ghrelin+ cell bodies had overlapping punctate CB1R+ signal (Fig. 10).

### Transcriptomic analysis

**Fig. 11:**
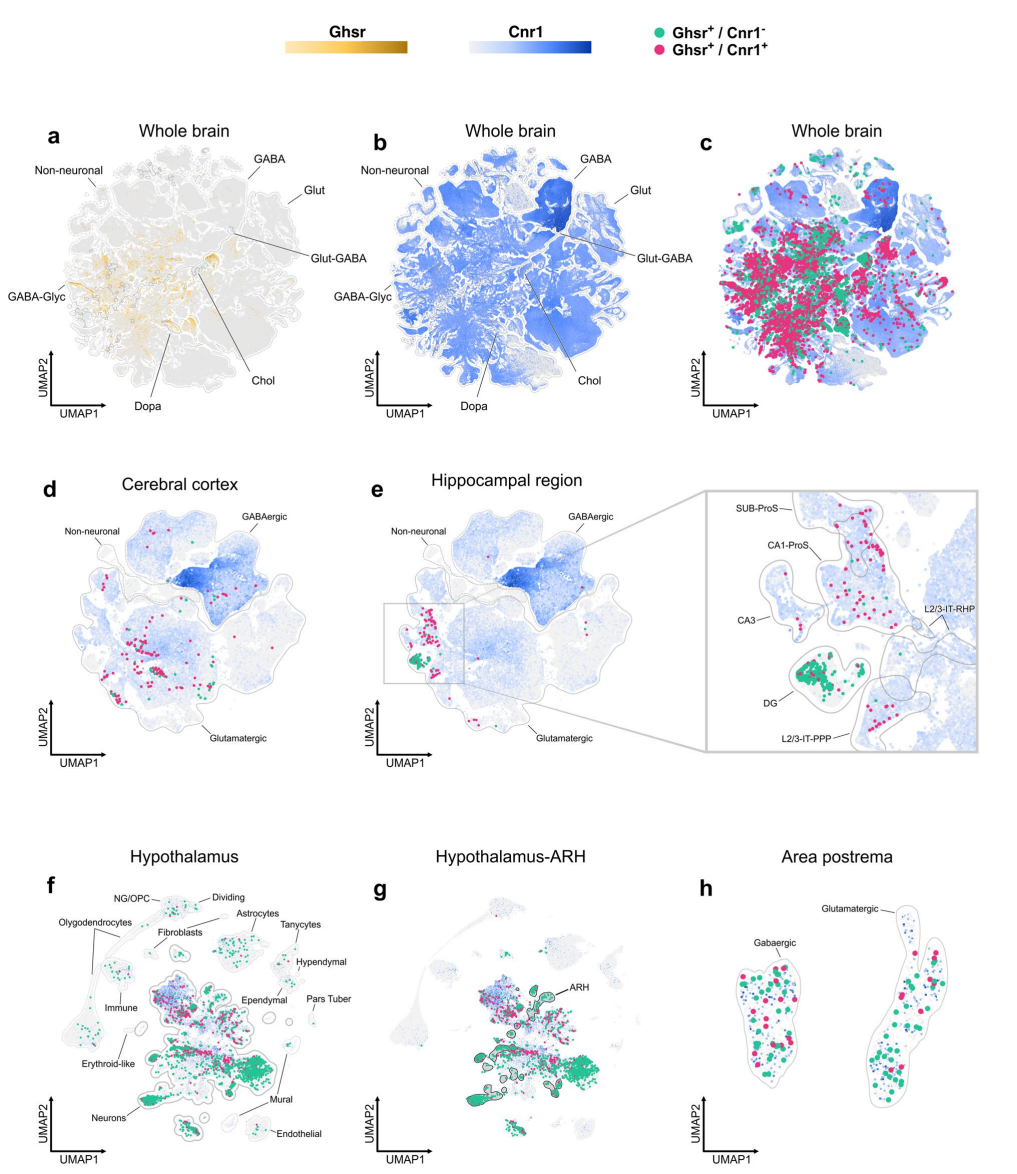
UMAP plots for the whole brain (**a**–**c**), cortex (**d**), hippocampal region (**e**), cells of the hypothalamus (**f**), neurons of the hypothalamus (**g**) and the area postrema (**h**) showing the gradient expression of Ghsr in gold or Cnr1 in a blue scale and the presence of single Ghsr+ (green circles) and double Ghsr+/Cnr1+ (purple circles) cells in the different clusters of each brain region. In each panel different cell types (**a**, **b**, **d**, **e**, **f** and **h**) or some specific regions (**e** and **g**) are delimited with black lines. Abreviations: Sub-ProS: prosubiculum proper-prosubiculum, CA1-ProS: Cornu Ammonis 1-prosubiculum, CA3: Cornu Ammonis 3, DG: dentate gyrus, L2/3-IT-PPP: layer 2/3-intratelencephalic region-parasubiculum/postsubiculum/presubiculum, L2/3-IT-RHP: layer 2/3-intratelencephalic region- retrohippocampal region and ARH: hypothalamic arcuate nucleus. UMAP coordinates and clusters were obtained from the corresponding original articles (Zhang et al. 2021; Yao et al. 2021, 2023; Steuernagel et al. 2022)

The analysis of the whole mouse brain scRNA-seq dataset indicated that the number of Cnr1+ cells is ~100-fold higher than the number of Ghsr+ cells (1,648,932 vs 16,425 cells, Fig. 11a, b). The dataset contained 9,554 Ghsr+/Cnr1+ double-positive cells, the majority of which were neurons (99.8%) and represented ~58.2% of all Ghsr+ cells but only ~0.6% of all Cnr1+ cells (Fig. 11c). Next, we performed a detailed analysis of the number, localization and phenotype of Ghsr+/ Cnr1+ neurons using the shared hierarchical taxonomy between the deep scRNA-seq dataset and the spatially resolved transcriptomic MERFISH dataset (Table 1). The quantitative analysis revealed that Ghsr+ cells were primarily located in the midbrain and in the hypothalamus, particularly in the ARH, whereas Cnr1+ neurons were widely distributed across various brain regions. Notably, Ghsr+/ Cnr1+ neurons were enriched in the midbrain (~28% of all Ghsr+/Cnr1+ neurons), with the majority including GABAergic and glutamatergic neurons in the PAG. The brain region with the second highest number of Ghsr+/ Cnr1+ neurons was the hypothalamus (~21% of all Ghsr+/ Cnr1+ neurons), with the highest concentration in the ARH (~15% of all hypothalamic Ghsr+/ Cnr1+ neurons) although most of them were found outside the ARH. In the ARH, these neurons represented 28.1% of all Ghsr+ cells and were primarily GABAergic. The area with the third highest number of Ghsr+/ Cnr1+ neurons was the hippocampal formation (~14 % of all Ghsr+/ Cnr1+ neurons), presenting glutamatergic neurons that represented 96.3, 47.1 and 29.8 % of all Ghsr+ neurons of the CA1, CA3 and DG. Hippocampal Ghsr+/Cnr1+ cells were enriched in the CAs regions, compared to the DG. Additionally, Ghsr+/ Cnr1+ neurons were found in the pontine reticular nuclei of the pons and several nuclei of the medulla, including NTS. In line with the neuroanatomical analysis, Ghsr+/ Cnr1+ neurons were found in the BLA and the MEA and they were almost exclusively glutamatergic. Only a few hundred of Ghsr+/Cnr1+ neurons were found in the cerebral cortex (not shown).

**Table 1:** Main results of the quantitative spatial transcriptomic analysis

Region [listed double+ cells/total double+ cells]	Structure name	positive cells (number)			double+ cells (%)							
		Ghsr	Cnr1	double	vs. Ghsr+ cells	vs. Cnr1+ cells	vs. neurotransmitter					
							GABA	GABA-Glyc	Glut-GABA	Glut	Chol	Dopa
Hippocampal formation (HPF) [4860/5561]	Field CA1 (CA1)	2500	212101	2407	96.3	1.1	0	0	0	100	0	0
	Field CA3 (CA3)	2801	70381	1320	47.1	1.9	1	0	0	99	0	0
	Dentate gyrus (DG)	3805	51506	1133	29.8	2.2	0	0	0	100	0	0
Cortical subplate (CTXsp) [1082/1747]	Basolateral amygdalar nucleus (BLA)	678	47985	629	92.7	1.3	0	0	0	100	0	0
	Posterior amygdalar nucleus (PA)	468	26537	453	96.8	1.7	0	0	0	100	0	0
Striatum (STR) [800/1523]	Medial amygdalar nucleus (MEA)	1693	43102	800	47.3	1.9	13	0	0	87	0	0
Pallidum (PAL) [441/1366]	Bed nuclei of the stria terminalis (BST)	647	22080	441	68.1	2.0	75	0	0	25	0	0
Hypothalamus (HY) [5712/8474]	Arcuate hypothalamic nucleus (ARH)	4437	7648	1247	28.1	16.3	76	0	1	8	12	3
	Dorsomedial nucleus of the hypothalamus (DMH)	634	8990	412	65.0	4.6	64	0	0	33	3	0
	Anterior hypothalamic nucleus (AHN)	662	23413	458	69.2	2.0	44	0	0	56	0	0
	Medial mammillary nucleus (MM)	1929	7144	431	22.4	6.0	0	0	3	97	0	0
	Medial preoptic nucleus (MPN)	602	15675	471	78.3	3.0	58	0	0	42	0	0
	Ventral premammillary nucleus (PMv)	549	8154	333	60.7	4.1	0	0	0	100	0	0
	Ventromedial hypothalamic nucleus (VMH)	1004	29262	729	72.6	2.5	3	0	0	93	3	1
	Posterior hypothalamic nucleus (PH)	627	17403	384	61.2	2.2	20	0	0	73	0	7
	Lateral hypothalamic area (LHA)	468	23427	341	73.0	1.5	67	0	0	30	0	4
	Zona incerta (ZI)	1274	41399	904	71.0	2.2	100	0	0	0	0	0
Midbrain (MB) [7535/10959]	Superior colliculus, sensory related (SCs)	551	19710	348	63.0	1.8	93	0	0	7	0	0
	Substantia nigra, reticular part (SNr)	1079	9846	580	53.8	5.9	100	0	0	0	0	0
	Ventral tegmental area (VTA)	2355	2167	332	14.1	15.3	39	0	0	0	0	61
	Midbrain reticular nucleus (MRN)	2011	36820	1408	70.0	3.8	48	3	5	45	0	0
	Superior colliculus, motor related (SCm)	1973	79564	1586	80.4	2.0	66	0	0	34	0	0
	Periaqueductal gray (PAG)	3663	97288	2669	72.9	2.7	52	3	1	40	0	3
	Pedunculopontine nucleus (PPN)	729	10680	613	84.1	5.7	9	2	0	83	2	5
Pons (P) [2387/4712]	Parabrachial nucleus (PB)	803	14388	708	88.2	4.9	3	0	3	88	0	6
	Pontine reticular nucleus, caudal part (PRNc)	1120	18550	856	76.4	4.6	0	69	0	31	0	0
	Pontine reticular nucleus (PRNr)	1004	23325	824	82.1	3.5	8	49	13	31	0	0
Medulla (MY) [3236/5245]	Area postrema (AP)	431	1618	377	87.5	23.3	0	100	0	0	0	0
	Nucleus of the solitary tract (NTS)	795	14017	778	97.8	5.5	0	71	0	20	9	0
	Gigantocellular reticular nucleus (GRN)	845	11060	474	56.2	4.3	0	49	0	51	0	0
	Intermediate reticular nucleus (IRN)	759	14177	538	70.9	3.8	0	62	0	38	0	0
	Parvicellular reticular nucleus (PARN)	769	16695	618	80.3	3.7	0	74	0	25	2	0
	Medial vestibular nucleus (MV)	1438	10581	450	31.3	4.3	2	61	0	37	0	0
Other [0/1129]												

Finally, we also estimated the co-expression of *Ghsr* and *Cnr1* in the mouse brain using additional scRNA-seq datasets derived from WT mice. In cortex and hippocampus (Yao et al. 2021), we found that *Cnr1* was highly expressed and enriched in GABAergic cells, whereas *Ghsr* showed a low level of expression and was mainly found in glutamatergic cells (Fig. 11d, e). In the cortex (Fig. 11d), 75.3% of Ghsr+ cells were also Cnr1+. All these cortical Ghsr+/Cnr1+ cells were identified as neurons, with 87% of them glutamatergic. In the hippocampal region (Fig. 11e), Ghsr*+* cells were mainly found in the DG (75.1%), as observed in the neuroanatomical studies, followed by the CAs regions (15.6%). Conversely, hippocampal Ghsr+/Cnr1+ cells were mainly distributed across the CAs regions (49.2%) and in less extent in the DG (23.3%), with 98.3% of them identified as glutamatergic neurons. Further analysis reveals that 93.7% of Ghsr+ neurons in the CA regions were Ghsr+/Cnr1+ cells, whereas only 9.2% of Ghsr+ neurons in the DG were Ghsr+/Cnr1+ cells. Within the hypothalamus (Steuernagel et al. 2022), 21% of all Ghsr+ cells are also Cnr1+ cells, with 98.4% of them being neurons (Fig. 11f). Ghsr+/Cnr1+ neurons were identified as either glutamatergic (47.8%) or GABAergic (52.2%) neurons (data not shown) and most of them were found outside the ARH (Fig. 11g). Notably, 58.5% of glutamatergic Ghsr+/Cnr1+ neurons were localized in the VMH, while 64.3% of GABAergic Ghsr+/Cnr1+ neurons were situated in the ARH. In the hypothalamus, Ghsr*+* cells included astrocytes, and most of them were Ghsr+/Cnr1- cells. In the AP (Zhang et al. 2021), 28.6% of the Ghsr+ cells were Ghsr+/Gnr1+ cells, with 61.5% of them identified as GABAergic and 38.5% of them identified as glutamatergic (Fig. 11h).

## Discussion

Our study presents the first analysis of the convergence of GHSR and CB1R signals in the mouse brain. We show that GHSR and CB1R primarily overlapped in specific neuronal subsets of the amygdala and hippocampus, where both receptors are co-expressed in glutamatergic neurons. GHSR+ cell bodies in the hypothalamus, midbrain, pons, and medulla also show significantly less, but still evident, overlap with CB1R signals, along with a lower degree of co-expression. Additionally, more varied types of neurons are found in most of these regions compared to the amygdala and hippocampus. These findings suggest that GHSR and CB1R differentially interact across specific mouse brain regions. This interaction may involve both simultaneous actions in certain neuronal populations and presynaptic modulation of GHSR+ neurons by CB1R, highlighting a complex interplay that could significantly influence neurophysiological processes. Of note, the number of Cnr1+ cells is approximately 100 times greater than that of Ghsr+ cells. Consequently, the small fraction of CB1R+ cells that co-express Ghsr may explain why the combined absence of both receptors in mice does not significantly impact food intake or body weight compared to mice lacking only one of the receptors (Mani et al. 2020).

### Methodological considerations

The GHSR-eGFP mouse strain was developed by the GENSAT project as part of effort to map the expression of thousands of genes across the mouse brain (Gong et al. 2003). This transgenic mouse line contains multiple copies of a modified *Ghsr* gene-containing bacterial artificial chromosome, in which the eGFP coding sequence is inserted at the translational start site of *Ghsr*, allowing the visualization of Ghsr-expressing cells by analyzing the presence of the fluorescent protein. We have validated the GHSR-eGFP mice using simultaneous *in situ* hybridization to detect of *Ghsr* mRNA and immunostaining to label eGFP, and shown that they specifically report *Ghsr* expressing cells in several brain nuclei, including the NTS, the parabrachial nucleus, the supramammillary nucleus and LHA (Mani et al. 2014; Cabral et al. 2017; Cornejo et al. 2018b; Aguggia et al. 2022; Barrile et al. 2023). Hence, GHSR-eGFP mice have played a crucial role in studying *Ghsr*-expressing cells, especially considering the lack of reliable anti-GHSR antibodies. Importantly, however, eGFP+ signal and *Ghsr* gene expression do not perfectly overlap in well-characterized brain regions of GHSR-eGFP mice and, consequently, some neuroanatomical observations must be interpreted with caution, as we have thoroughly discussed in the past (Mani et al. 2014). For instance, GHSR-eGFP mice have few eGFP+ cells in other brain regions, such as the VTA, SNr, and hypothalamic nuclei, including the ARH, where *Ghsr* gene expression has been consistently reported (Zigman et al. 2006; Chuang et al. 2011; Scott et al. 2012). Conversely, GHSR-eGFP mice exhibit a high number of eGFP+ cells in regions such as the olfactory bulbs, cerebral cortex, and amygdala, where Ghsr mRNA was not detected using radioactive *in situ* hybridization (Zigman et al. 2006). Consequently, the accuracy of the eGFP+ signal as an indicator of Ghsr expression in these brain areas of this mouse model could not be confirmed using dual-label *in situ* hybridization and immunohistochemistry (Mani et al. 2014). Some evidence, however, suggests that Ghsr is expressed in the olfactory bulbs of the mouse brain, where biotin-ghrelin binding was reported (Al Massadi et al. 2011). In the amygdala, *Ghsr* gene expression or function was reported in mouse and rat BLA (Walker et al. 2012; Alvarez-Crespo et al. 2012) and central nuclei of rat amygdala (Landgren et al. 2011; Cruz et al. 2013). Also, eGFP+ cells are also observed in the amygdala, entorhinal cortex and olfactory tubercle of another Ghsr-IRES-tauGFP reporter mice (Jiang et al. 2006). Thus, eGFP+ cell bodies in GHSR-eGFP mice appear to accurately represent Ghsr-expressing cells in some, but not all, of the brain regions mapped in this study.

The use of Fr-ghrelin provided a complementary map of the presence of GHSR+ cells, as we previously shown (Cabral et al. 2013; Fernandez et al. 2016, 2023; Cornejo et al. 2018a; Uriarte et al. 2021; Aguggia et al. 2022). Fr-ghrelin is a variant of ghrelin constituted by the bioactive N-terminal end of the hormone conjugated to a far-red-fluorescent dye that can be directly imaged in the samples (Barrile et al. 2019; Uriarte et al. 2021). Fr-ghrelin binds to GHSR and induces intracellular inositol phosphate accumulation in *Ghsr*-expressing HEK293T cells to a similar extent as ghrelin (Leyris et al. 2011). Additionally, Fr-ghrelin stimulates food intake like ghrelin and labels cells in all *Ghsr*-expressing periventricular brain areas in WT mice, but not in GHSR-deficient mice (Uriarte et al. 2021). *In vivo*, Fr-ghrelin labeling allows for the direct identification of cells containing GHSR protein capable of binding ghrelin, in contrast to c-Fos immunostaining, which labels cells that are either directly or secondarily activated by the hormone (Fernandez et al. 2018). Since Fr-ghrelin poorly labels neuronal fibers, the transport of GHSR from the cell bodies to terminals may be a factor that adds discrepancies between cells identified as GHSR+ neurons in each experimental strategy. Additionally, centrally infused Fr-ghrelin depicts a limited penetration into the brain from the ventricular wall, and consequently mainly labels GHSR+ cells located in periventricular regions (Cabral et al. 2014, 2015; Uriarte et al. 2019). Of note, Fr-ghrelin also labels cells in regions not directly adjacent to the fourth ventricle of the caudal medulla, such as the NTS and DMV, as reported previously (Cornejo et al. 2018b), likely due to the permeable nature of the blood-brain barrier in this part of the brain (Maolood and Meister 2009). Finally, it is worth considering that Fr-ghrelin can be internalized by cells that lack *Ghsr* mRNA, such as the hypothalamic tanycytes (Uriarte et al. 2021), presumably via pathways aimed to clear extracellular peptides. These properties of Fr-ghrelin labeling also must be considered during the analysis of the current experimental outcomes.

The presence of CB1R was visualized using an antiserum raised against the C-terminal end of CB1R that specifically labels this receptor, as indicated by the lack of labeling in CB1R-knock out mice (Hájos et al. 2000; Wager-Miller et al. 2002). Our findings on the neuroanatomical distribution of CB1R+ signal in the mouse brain align with previous rodent studies, showing higher intensity in cortical structures like the hippocampus and the amygdala, and lower detection in the hypothalamus or brainstem (Tsou et al. 1998; Nyíri et al. 2005; Wittmann et al. 2007; Cardinal et al. 2014; Davis et al. 2018; Shen et al. 2019). Such scarce CB1R+ labeling in the brain regions described in the results section may result from the transport of CB1R from cell bodies to distantly located terminal axons and presumably limited our capability to unmask cells expressing both GPCRs. The CB1R+ signal in the rodent brain has also been extensively characterized at the cellular level (Tsou et al. 1998; Katona et al. 1999). In this regard, CB1R+ signal can exhibit a somatodendritic pattern, observed in different brain areas, such as cerebral cortex, hippocampus, or amygdala (Katona et al. 1999; Marsicano and Lutz 1999). This pattern corresponds to receptor located in intracellular compartments of the soma and proximal dendrites, such as rough endoplasmic reticulum, Golgi apparatus and even mitochondria (Katona et al. 1999; Bénard et al. 2012; Lutz 2020). CB1R+ signal can also display a punctate pattern, which reveals the presence of presynaptic receptor in axon terminals as indicated by using post-embedding immunogold method in electron microscopy (Nyíri et al. 2005), and has been reported in different brain areas such as the hypothalamus (e.g., ARH), midbrain (e.g., VTA), hippocampus (e.g., CA) or medulla (e.g., NTS) (Katona et al. 1999; Melis et al. 2004; Seagard et al. 2004; Osei-Hyiaman et al. 2005). Here, we observed eGFP+ or Fr-ghrelin+ cell bodies exhibiting overlapping and perisomatic CB1R+ signal in various brain areas. Unfortunately, the resolution limit in fluorescence microscopy prevented us from determining with certainty if such double labeling corresponds to somatodendritic CB1R or presynaptic CB1R located in axons that innervate GHSR+ cells. As discussed below, the co-expression of both GPCR in the same cells was further estimated based on the transcriptomic dataset analysis. Conversely, the extent to which presynaptic CB1R regulates GHSR+ neurons in the mouse brain could not be precisely determined in this study. Presynaptic CB1R mediates retrograde signaling, which involves the action of postsynaptically released endocannabinoids that travel backwards across the synapse to regulate neurotransmitter release, and constitutes the primary mechanism through which CB1R mediates synaptic plasticity (Castillo et al. 2012). Thus, endocannabinoid retrograde signaling may serve as a key mechanism by which GHSR+ cells and CB1R+ terminals interact; however, determining the neuronal populations where this pathway occurs requires further detailed study.

The transcriptomic analyses served as a complementary strategy to estimate the proportions of cells co-expressing *Ghsr* and *Cnr1* genes. The single-cell RNA-seq dataset analysis revealed an overall expression pattern of these receptors consistent with previous studies that assessed the independent distribution of Ghsr or Cnr1 mRNA using more traditional techniques (Katona et al. 1999; Lein et al. 2007), although it may have led to underestimate the number of *Ghsr*-expressing cells due to the lower gene expression/copy number of *Ghsr* compared to *Cnr1*. Of note, the complex molecular mechanisms governing the gene translation of these receptors and the localization of the resulting proteins within specific cellular compartments may cause apparent discrepancies or require special considerations during the simultaneous analysis of imaging and transcriptomic results. For instance, the *Ghsr1* gene encodes two isoforms: GHSR1a, the functional receptor that mediates ghrelin effects, and GHSR1b, which does not bind ghrelin (Gnanapavan et al. 2002). The mouse *Cnr1* gene encodes one CB1R isoform (Miller and Devi 2011); however, the intensity of CB1R+ signal does not always correlate with *Cnr1* mRNA levels assessed using *in situ* hybridization, a discrepancy attributed to the high trafficking of the receptor to terminals, where the protein accumulates (Mackie 2005). Despite these shortcomings, we consider that the joint observation of cells expressing both *Ghsr* and *Cnr1* genes in the transcriptomic analysis, along with the observation of eGFP+ and Fr-ghrelin+ cells with robust overlapping CB1R+ signals in the neuroanatomical studies, provides a reasonable indication that specific neuronal subsets simultaneously possess both receptors.

### Neuroanatomical and functional considerations

Considering the above points, the current results help to unmask specific areas in the mouse brain where the actions of GHSR and CB1R converge. Given the polymorphic nature of the pattern of the CB1R+ signal in different brain areas, it seems likely that the extent and relevance of the CB1R and GHSR crosstalk depends on each particular brain area.

We found here that GHSR+ cell bodies located in specific regions such as the hippocampus and amygdala were intermingled in a meshwork of CB1R+ fiber-like structures, whose intensity and density depended on the specific brain area. In the hippocampus, eGFP+ and Fr-ghrelin+ cell bodies were mainly clustered in the base of the DG-sg, where excitatory dentate granular cells are enriched. However, most GHSR+ cells in the DG did not show overlapping CB1R+ signal, which was observed in fiber-like structures clustered in the top of DG-sg, proximal to the cell bodies. Transcriptomic dataset indicated that only a fraction of Ghsr+ cells in the DG simultaneously express *Cnr1*. Thus, direct crosstalk between both GPCRs appears to occur in specific glutamatergic cells of the DG, although it may not be their primary mode of interaction in this brain area. In the pyramidal layer of the CA3, GHSR+ cell bodies were found to be scarce and intertwined within a densely packed meshwork of CB1R+ fiber-like structures. Here, most GHSR+ cell bodies had overlapping CB1R+ signal, in both GHSR-eGFP mice and mice injected with Fr-ghrelin. Transcriptomic analysis revealed a high fraction of *Ghsr*-expressing cells in the CAs and subiculum regions simultaneously express *Cnr1*, and that hippocampal cells co-expressing these receptors were glutamatergic neurons. Previous studies have shown that GHSR and CB1R signaling mainly target GABAergic neurons in the hippocampus. For instance, GHSR deletion was shown to suppress the intrinsic excitability of GABAergic interneurons dCA1, but not of neighbouring pyramidal neurons (Li et al. 2021, 2023). CB1R+ signal, in turn, was shown to mainly label GABAergic basket cells, a subset of hippocampal GABAergic interneurons that surround cell bodies of pyramidal cells (Nyíri et al. 2005; Lee et al. 2010), but not excitatory neurons such as dentate granule cells or pyramidal neurons (Katona et al. 1999; Marsicano and Lutz 1999). Indeed, CB1R was found to be enriched in the presynaptic terminals of hippocampal GABAergic interneurons and control GABA release (Tsou et al. 1998; Katona et al. 1999, 2001; Hájos et al. 2001). However, CB1R mRNA was also detected in non-GABAergic cells in the hippocampus (Marsicano and Lutz 1999), where CB1R agonists suppress excitatory transmission (Kawamura et al. 2006). Thus, it is likely that crosstalk between the GHSR and CB1R systems include simultaneous action in glutamatergic hippocampal neurons as well as other varied mechanisms to influence cognitive functions, such as learning or memory, which are strongly affected by these GPCRs in rodents (Reibaud et al. 1999; Marsicano et al. 2002; Diano et al. 2006).

Similar to CA3, GHSR+ cells in the BLA or PAA of the amygdala were intermingled in an overlapping loose packed meshwork of CB1R+ fiber-like structures. Here, we provide the first evidence that both receptors are co-expressed in glutamatergic amygdalar neurons, in line with previous studies showing that ghrelin indirectly inhibits large, but not small, pyramidal-like neurons by reducing mEPSCs frequency (Alvarez-Crespo et al. 2012). Furthermore, perfusion of ghrelin increased the activity of inhibitory GABA inputs into central amygdala neurons (Cruz et al. 2013). Conversely, CB1R is known to be highly expressed in GABAergic interneurons of the amygdala and to reduce inhibitory tonic control over local pyramidal cells (Katona et al. 2001). Thus, it seems likely that GHSR and CB1R signalling converges in interneurons of the amygdala and such crosstalk could impact on memory-, reward-, and/or stress-related processes, all of which are regulated by both GPCRs (Brunt & Bossong 2022; Cornejo et al. 2021a, b). Supporting this possibility, we found that a subset of GABA neurons in the MEA co-expresses both receptors.

In the cerebral cortex, neuroanatomical analysis showed GHSR+ cells intermingled within a CB1R+ fiber-like meshwork, looser than in the hippocampus, but still overlapping with putative GHSR+ cells. Transcriptomic analysis revealed that Cnr1 is highly express in the cerebral cortex and enriched in GABAergic cells, in line with previous findings showing that CB1R is mainly found in cortical GABAergic interneurons (Busquets-Garcia et al. 2018). Conversely, transcriptomic analysis indicated that a small subset of cerebral cortex neurons expresses *Ghsr*. GHSR+ neurons do appear to exist in the cerebral cortex, as ghrelin can act on cortical neurons (Mir et al. 2018). However, the number of these cells is extremely small, with no evident signal detected using *in situ* hybridization analysis (Zigman et al. 2006), and it seems likely that GHSR-eGFP mice overestimate the number of GHSR+ cells in this brain region. Thus, GHSR and CB1R may interact in the cerebral cortex, potentially involving a subset of cortical Ghsr+/Cnr1+ glutamatergic neurons, as suggested by transcriptomic analysis. However, the extent of their functional interaction in the cerebral cortex is likely minor, and the physiological implications remain to be studied.

GHSR and CB1R were also found in the GPi. Here, we confirmed that *Ghsr*-expressing cells are present in the mouse GPi, as previously reported (Mani et al. 2014). In line with this observation, ghrelin treatment was shown to reduce glucose metabolism in the GPi, but the functional implications of such an action remain uncertain (De Francesco et al. 2022). As observed in rats (Tsou et al. 1998), we also found that the CB1R antibody intensely labels the GPi. Previous studies have revealed that CB1R in the GPi is located in fibers that densely innervate this brain region and correspond to medium spiny neuron axons and terminals involved in the modulation of the neural circuits that control voluntary movement (Tsou et al. 1998; Engler et al. 2006). Further studies are required to clarify the role and functional implications of these CB1R+ terminals in GHSR+ neurons within the GPi.

Current neuroanatomical studies have also revealed the presence of scarce and weak CB1R+ signal in the hypothalamus (e.g., ARH), midbrain (e.g., VTA), and medulla oblongata. The CB1R+ signal consists of punctate structures that are typically small, round, closely spaced, and sometimes even overlapping with eGFP+ and Fr-ghrelin+ cell bodies. The reduced amount of CB1R in these brain areas, relative to other brain regions, aligns with earlier findings based on the analysis of binding of a radiolabeled cannabinoid (Herkenham et al. 1990). CB1R is known to be located in presynaptic axon terminals, where it can potently regulate neurotransmission in the above listed brain areas (Katona et al. 1999; Melis et al. 2004; Seagard et al. 2004; Wittmann et al. 2007). The presynaptic action of CB1R in different areas of the forebrain and the brainstem is well established and it is likely to affect targeted GHSR+ cells. Unlike in the hippocampus or cerebral cortex where CB1R is enriched in GABAergic terminals (Kawamura et al. 2006), CB1R is found in both symmetric and asymmetric synapses of hypothalamic nuclei (Wittmann et al. 2007). Thus, presynaptic CB1R likely modulates inhibitory and excitatory neurotransmission on subsets of neurons expressing somatic GHSR that exist in different nuclei of the hypothalamus, such as the ARH (van den Top et al. 2004). In addition, it is interesting to highlight that transcriptomic analysis indicated that cells co-expressing *Ghsr* and *Cnr1* are mainly located outside the ARH and that hypothalamic neurons co-expressing *Ghsr* and *Cnr1* are not only glutamatergic but also GABAergic, with the former mainly localized in the VMH and the latter in the ARH. Thus, GHSR and CB1R cross-talk in the hypothalamus seems to be complex and will vary depending on the specific region and cell type. Transcriptomic analysis also indicated that different, though specific, types of *Ghsr*-expressing cells exhibit *Cnr1* expression in the midbrain and medulla. For instance, the transcriptomic analysis indicated that Ghsr+/Cnr1+ cells in the VTA mainly include dopaminergic but also GABA cells. Previous studies showed that CB1R in the VTA acts on GABAergic and glutamatergic terminals to modulate the activity of the dopamine neurons (Riegel and Lupica 2004), whereas GHSR is expressed in dopaminergic and GABA neurons in the VTA (Abizaid et al. 2006; Cornejo et al. 2020). Thus, GHSR and CB1R seem to simultaneously act via multiple mechanisms to regulate this key center in reward-related processes. Intriguingly, the largest number of Ghsr+/Cnr1+ cells was found in the PAG, a midbrain area that shows few GHSR+ cells in the GHSR-eGFP mice (Mani et al. 2014) and limited CB1R immunolabeling, highlighting the need for further investigation. In the AP, transcriptomic analysis showed discrepancies: one dataset indicated *Ghsr* and *Cnr1* expression is restricted to GABA/glycinergic cells, while another suggested they are expressed in GABAergic and glutamatergic neurons. Further experimental validation is necessary to clarify the precise neuronal populations expressing these receptors and to determine the physiological significance. In the medulla oblongata, CB1R was also found to suppress GABA and glutamate transmission in neurons of the DMNV and the NTS (Derbenev et al. 2004; Roux et al. 2009), centers known to be enriched GSHR+ neurons (Cornejo et al. 2018b). Here, we found that Ghsr+/Cnr1+ cells also exist in the NTS and are primarily GABA/glycinergic cells. Thus, simultaneous interactions in the same cell types, along with the presynaptic action of CB1R on GHSR+ neurons, may contribute to fine-tuning the influence of these receptors on various central functions, including food intake, neuroendocrine axis activity, gastrointestinal function, and reward processing, all of which are regulated by these GPCRs in the said brain areas (Mechoulam and Parker 2013; Cornejo et al. 2021b).

The polymorphic CB1R+ staining pattern found in the mouse brain includes the visualization of highly stained CB1R+ cell bodies, primarily located in the cerebral cortex, the hippocampus and basolateral amygdala. Notably, no overlap was found between these distinct CB1R+ cell bodies and the GHSR+ signal. Although the identity of the CB1R+ cell bodies was not confirmed, transcriptomic analysis strongly suggests that they include GABAergic interneurons, as discussed above. As previously described in rats (Tsou et al. 1998), we also observed a unique CB1R+ staining pattern in the SNr and globus pallidus, characterized by a dense, fine, unbeaded fiber meshwork. Strikingly, GHSR+ cells in the SNr showed overlapping CB1R+ signal, whereas GHSR+ cells in the globus pallidus were observed intermingled within CB1R+ signal, displaying a punctate pattern. In line with these observations, several studies have shown that cannabinoids and ghrelin modulate the activity of striato-nigral, striato-pallidal and subthalamo-nigral pathways (Antonazzo et al. 2019; De Francesco et al. 2022). However, the extent to which these GPCR systems engage in cross-talk at a functional level, and the implications of such different types of neuroanatomical interactions, remain to be explored.

Some evidence indicates that the *Ghsr* and *Cnr1* genes are expressed in glial cells. CB1R has been found in astrocytes from several brain regions, including the hippocampus (Castillo et al. 2012) and the caudate putamen (Rodriguez et al. 2001). Similarly, GHSR has been found in hypothalamic astrocytes, which respond to ghrelin treatment (Fuente-Martín et al. 2016). Here, we cannot provide any conclusion on whether astrocytes can directly integrate GHSR and CB1R signaling, as we did not use any markers of glial cells in the neuroanatomical studies. However, we found this possibility unlikely since transcriptomic analysis indicates that the vast majority of cells co-expressing *Ghsr* and *Cnr1* genes are neurons.

### Conclusion

This study, we provide new insights into the specific neuronal subset of the mouse adult brain areas where GHSR and CB1R systems interact. It remains to be studied whether expression patterns vary across sex or different developmental stages and the extent to which these findings apply to the crosstalk and function of GHSR and CB1R in the human brain. Current findings may open new avenues for innovative dual-target therapeutic strategies aimed at addressing metabolic disorders, such as obesity, or psychiatric conditions.

## Data Availability

The data supporting the findings of this study, as well as key reagents, are available upon reasonable request to the corresponding authors, PNDF or MP. Data and materials supporting the results or analyses presented in the paper are available upon reasonable request
